# Primary tumor resection: a new hope or an old illusion for patients with metastatic non-small cell lung neuroendocrine tumors?

**DOI:** 10.1186/s12957-025-04063-y

**Published:** 2025-10-31

**Authors:** Hongquan Xing, Weichang Yang, Shanshan Cai, Linmin Xiong, Guofeng Zhu, Xinyi Zhang, Xiaoqun Ye

**Affiliations:** 1 Department of Respiratory and Critical Care Medicine, The Second Affiliated Hospital, Jiangxi Medical College, Nanchang University, Nanchang, 330006 China; 2 Jiangxi Key Laboratory of Molecular Medicine, The Second Affiliated Hospital of Nanchang University, Nanchang, 330006 China; 3 Department of Thoracic Surgery, The Second Affiliated Hospital, Jiangxi Medical College, Nanchang University, Nanchang, 330006 China

**Keywords:** Metastatic non-small cell neuroendocrine tumor, Machine learning, SEER, Primary tumor resection, Nomogram

## Abstract

**Objectives:**

This study aimed to investigate the impact of primary tumor resection (PTR) on survival outcomes for patients with metastatic non-small cell neuroendocrine tumors (mNSCLC-NETs), develop a predictive model to identify which patients may benefit from surgery in terms of survival.

**Methods:**

We extracted information on mNSCLC-NET patients from the SEER database. Propensity score matching was used to eliminate bias between surgery and non-surgery groups. The effect of PTR on prognosis was assessed via Kaplan‒Meier analysis with the log-rank test and the Cox proportional hazards model. Feature selection was performed via the Boruta algorithm. Model building utilized fivefold cross-validation and applied five machine learning algorithms. The optimal model was selected and used to construct a visual network nomogram.

**Results:**

Among the 1,776 eligible patients, 12.61% underwent surgery. After PSM, the surgery group showed significantly longer median overall survival (mOS) (26 months vs. 11 months) compared to the non-surgery group. Among the five machine learning models, logistic regression had the highest AUC of 0.760 on the validation set. Therefore, we used a logistic regression model to construct a nomogram. This tool identified beneficiary and non-beneficiary groups, with the former having a longer mOS (30 months vs. 10 months).

**Conclusions:**

Overall, PTR in mNSCLC-NETs could prolong patients survival, and the web-based nomogram can predict patients who may benefit from surgery. This tool may aid clinicians in patient counseling and personalized decision-making.

**Supplementary Information:**

The online version contains supplementary material available at 10.1186/s12957-025-04063-y.

## Introduction

Pulmonary neuroendocrine tumors, including small cell lung cancer (SCLC) and non-small cell neuroendocrine tumors (NSCLC-NETs), constitute approximately 25% of primary lung cancers [1]. While SCLC incidence has declined, NSCLC-NETs have increased by 3% annually [2], posing a growing challenge to healthcare. For early-stage NSCLC-NET patients, surgical resection is usually the preferred treatment method [3]. However, approximately one-fifth of patients have distant metastasis at the time of initial diagnosis [4], and the overall survival (OS) rate for those in the metastatic stage is approximately 27% over five years [5]. Moreover, cases of metastatic non-small cell neuroendocrine tumors (mNSCLC-NETs) are typically considered incurable, with the treatment goal primarily focusing on managing symptoms and enhancing quality of life; thus, primary tumor resection (PTR) is rarely used as a standard treatment [6]. However, due to PTR, patients with advanced non-small cell lung cancer (NSCLC) experience increased survival rates [7]. Furthermore, previous studies have suggested that PTR may benefit select patients with mNSCLC-NETs [8, 9]. Nevertheless, the small sample sizes limit these studies. Moreover, due to significant individual differences among neuroendocrine tumor patients, postoperative outcomes can vary greatly [10]. To date, there are no clear standards for accurately predicting which mNSCLC-NET patients may benefit from PTR. If clinicians can perform risk stratification for lung neuroendocrine tumor patients and accurately predict their prognosis, more attention can be given to high-risk patients, and appropriate measures can be taken in advance.

In recent years, machine learning (ML), a new type of artificial intelligence, has been increasingly used for cancer detection and predictive data analysis [11]. Therefore, we compared various ML algorithms with traditional statistical methods (logistic regression (LR)) and used extensive data from the SEER database to design a personalized nomogram while also developing a web-based tool. Our goal was to create a predictive model to support the identification of which mNSCLC-NET patients may benefit from PTR.

## Materials and methods

### Patients

The Surveillance, Epidemiology, and End Results (SEER) database covers data pertaining to approximately 28% of cancer patients in the United States [12]. We used SEER*Stat 8.4.3 to identify NSCLC-NET patients from the SEER 17 Regs database with data from November 2023. We used the 8th edition of the American Joint Committee on Cancer (AJCC) to reclassify the TNM stages and generate a uniform dataset.

Our criteria for evaluating patients were as follows: (i) diagnosis confirmed by histology and stage IV; (ii) International Classification of Diseases for Oncology, Third Edition (ICD-O-3) codes 8013/3, 8240/3, 8249/3, and one primary malignant lung tumor only (C34x); and (iii) diagnosis between 2000 and 2021.

**Fig. 1:**
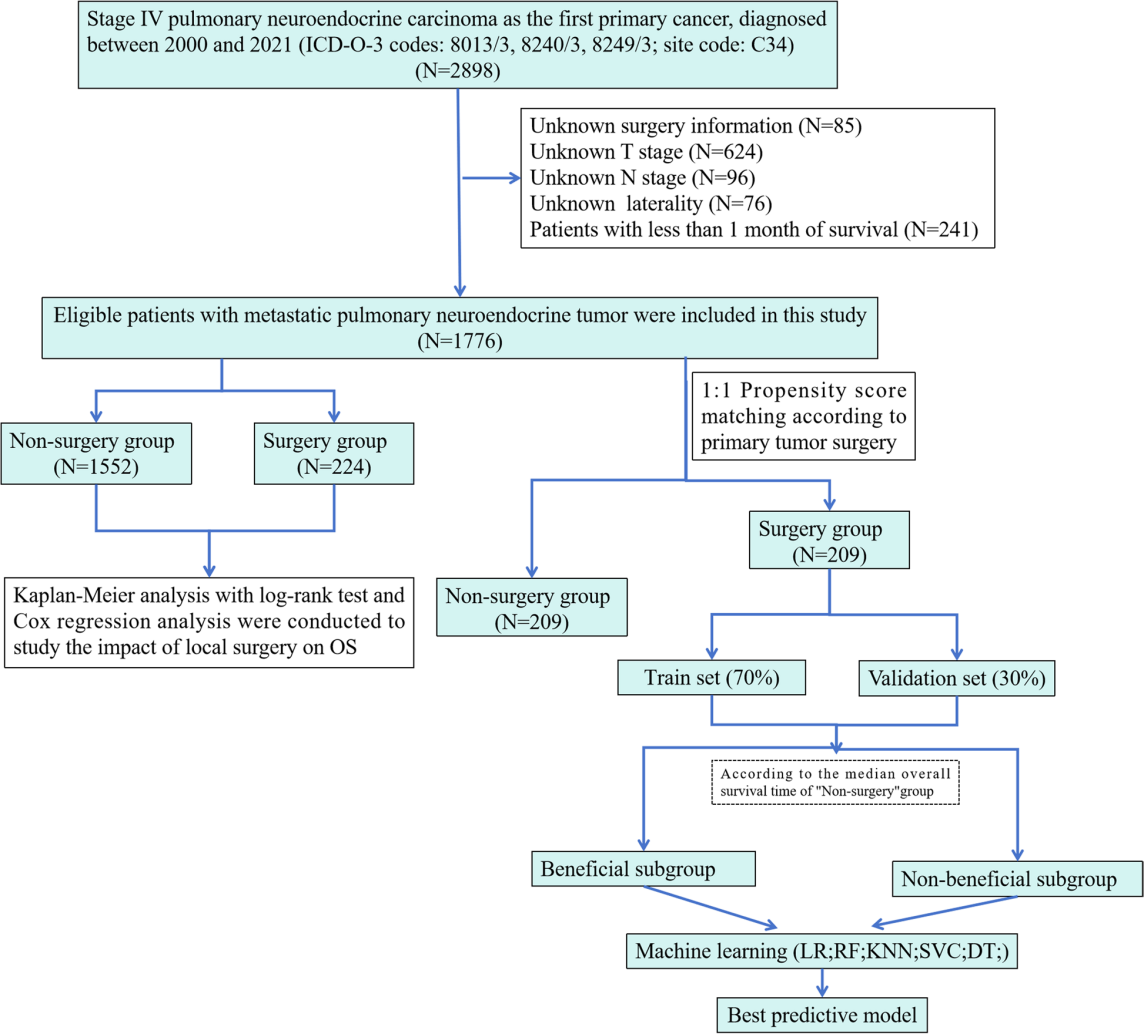
Flow chart of patient enrollment

The exclusion criteria were as follows: (i) unknown surgery information; (ii) unknown T/N stage and laterality; and (iii) survival time of less than one month. The inclusion and exclusion criteria are presented in Fig. 1.

The variables obtained from the SEER database included the year of diagnosis, age (≤ 65 years, > 65 years), sex, race, laterality (right, left), primary site, T stage, N stage, AJCC stage, radiation recode, chemotherapy, PTR, survival months and vital status records. The follow-up time was defined as the span between the diagnosis of NSCLC-NETs and the occurrence of death, the last follow-up, or the end of the follow-up period (December 31, 2021).

### Statistical analysis

To allow for nonlinear relationships, age was treated as a continuous variable and categorized after evaluation via a restricted cubic spline function [13]. To minimize confounding bias and match patients between the two groups, we applied propensity score matching (PSM) at 1:1 ratio. Matching was based on the nearest propensity scores on the logit scale, using a caliper of 0.1. We performed a power evaluation using the R package PSpower [14] on the unmatched cohort (*N* = 1776). The results indicated that, under the clinically more relevant Average Treatment Effect on the Treated (ATT) and Average Treatment Effect in the Overlap Population (ATO) frameworks, the current sample size provided adequate statistical power (> 80%). We employed the chi-square test or Fisher’s exact test to assess differences in selected variables between the two groups, both before and after PSM analysis. Next, we compared overall survival (OS) between the initial and matched groups via Kaplan‒Meier (K‒M) analysis and the log-rank test. Moreover, we applied univariate and multivariate Cox proportional hazards models to assess the prognostic value of PTR.

### Development and validation of a predictive nomogram model

Based on the post-PSM cohort, we defined surgical beneficiaries as patients with mNSCLC-NETs who underwent PTR and had a longer median OS (mOS) than those who did not undergo surgery. The Boruta algorithm identifies significant features by comparing feature Z values with shadow feature Z values through random forest model iterations [15] and is applied to select feature parameters before model construction. For the post-PSM cohort, surgery group participants were randomly split into a training set (70%) and a validation set (30%) for analysis. To achieve the highest prediction performance, five models were built, including the LR, random forest (RF), decision tree (DT), support vector machine (SVC), and K-nearest neighbor (KNN) models. In the training dataset, the model was validated through fivefold cross-validation. Thirty percent of the original dataset was used as a test set for evaluating the performance of the model. The LR model was transformed into a nomogram to understand and use the model as a quantitative tool for predicting which mNSCLC-NET patients might benefit from PTR. We assessed the model’s predictive performance in both the training and validation sets via the area under the receiver operating characteristic curve (AUC). The Hosmer–Lemeshow test and a calibration curve generated from 1,000 repeated samples were used to examine the consistency between the model and real-world situations. The clinical usefulness of the model was assessed through decision curve analysis (DCA), which evaluates the net benefit of intervention measures based on the model.

A risk score was calculated for each patient via this model, and the median risk score was used to categorize patients into those who would benefit from surgery and who would not. A K‒M survival curve was used to demonstrate the model's ability to differentiate between patients who would benefit from PTR (surgery benefit-candidates vs. surgery no-benefit-candidates vs. no-surgery group). Finally, a dynamic web-based nomogram is published for the reader's use. We used Empower Stats (www. empowerstats.com) and R (http://www.R-project.org) for all the analyses. The acceptable level of statistical significance was < 0.05.

## Results

### Patient characteristics

Our study included 1776 eligible patients with mNSCLC-NETs between 2000 and 2021. Table 1 summarizes the demographic and pathological characteristics of the patients. Among them, many patients were male (*n* = 928, 52.25%), White (*n* = 1445, 81.93%) and had right laterality (*n* = 1058, 59.57%). Most of the normal sites were the upper lobes (*n* = 829, 46.68%), followed by the lower lobes and other regions. PTR was performed in 224 patients (12.61%), chemotherapy in 1057 patients (59.52%) and radiation therapy in 787 patients (44.31%).

**Table 1:** Basic clinicopathological characteristics of the included patients with mNSCLC-NET

Variables	Total (*n* = 1776)
Age (%)	
≤ 65 years	911 (51.30)
> 65 years	865 (48.70)
Sex (%)	
Female	848 (47.75)
Male	928 (52.25)
Race (%)	
White	1455 (81.93)
Others	321 (18.07)
Marital (%)	
Married	931 (52.42)
Not married	845 (47.58)
Histology (%)	
Typical carcinoid	346 (19.48)
Atypical carcinoid	165 (9.29)
LCNEC	1265 (71.23)
Laterality (%)	
Right	1058 (59.57)
Left	718 (40.43)
Primary Site (%)	
Upper lobe	829 (46.68)
Middle lobe	134 (7.55)
Lower lobe	516 (29.05)
Others	297 (16.72)
T stage (%)	
T1-2	770 (43.36)
T3-4	1006 (56.64)
N stage (%)	
N0	545 (30.69)
N1-3	1231 (69.31)
Radiation (%)	
No	989 (55.69)
Yes	787 (44.31)
Chemotherapy (%)	
No	719 (40.48)
Yes	1057 (59.52)
Surgery (%)	
No	1552 (87.39)
Yes	224 (12.61)

*Abbreviations*: *mNSCLC-NET* metastatic non-small cell neuroendocrine tumor, *LCNEC* Large cell neuroendocrine carcinoma

### Prognosis of each treatment modality

**Fig. 2:**
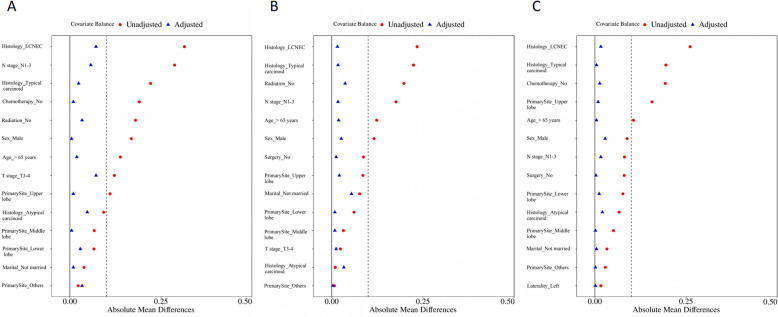
Standardized mean differences before and after PSM. **A** 1:1 match of patients with PTR and those without PTR; **B** 1:1 match of patients with chemotherapy and those without chemotherapy; **C** 1:1 match of patients with radiation therapy and those without radiation therapy

PSM was employed for each treatment approach administered to the patients. We established 1:1 matched cohorts for different treatment comparisons. Specifically, we compared the surgery cohort (*n* = 209) with the non-surgery group (*n* = 209), the radiotherapy cohort (*n* = 677) with the non-radiotherapy group (*n* = 677), and the chemotherapy cohort (*n* = 570) with the non-chemotherapy group (*n* = 570). In these matched cohorts, all variables were effectively balanced (*P* > 0.05 and SMD < 0.1; see Tables S1 - S3and Fig. 2).

Before PSM, the Cox regression model was used to adjust for potentially confounding factors that were not fully balanced (Tables S4). The adjusted KM analysis revealed that there was no statistically significant difference in the mOS between patients who received radiotherapy and those who did not (mOS: 7 months vs. 9 months, *P* > 0.05). Conversely, undergoing surgery and chemotherapy significantly enhanced the patients’ survival probability (*P* < 0.05, Figure S1). The mOS of patients in the surgery receiving group and the chemotherapy receiving group were 27 months and 9 months, respectively, which exceeded that of patients in the non—surgery and non—chemotherapy groups (7 months and 5 months, respectively).

**Fig. 3:**
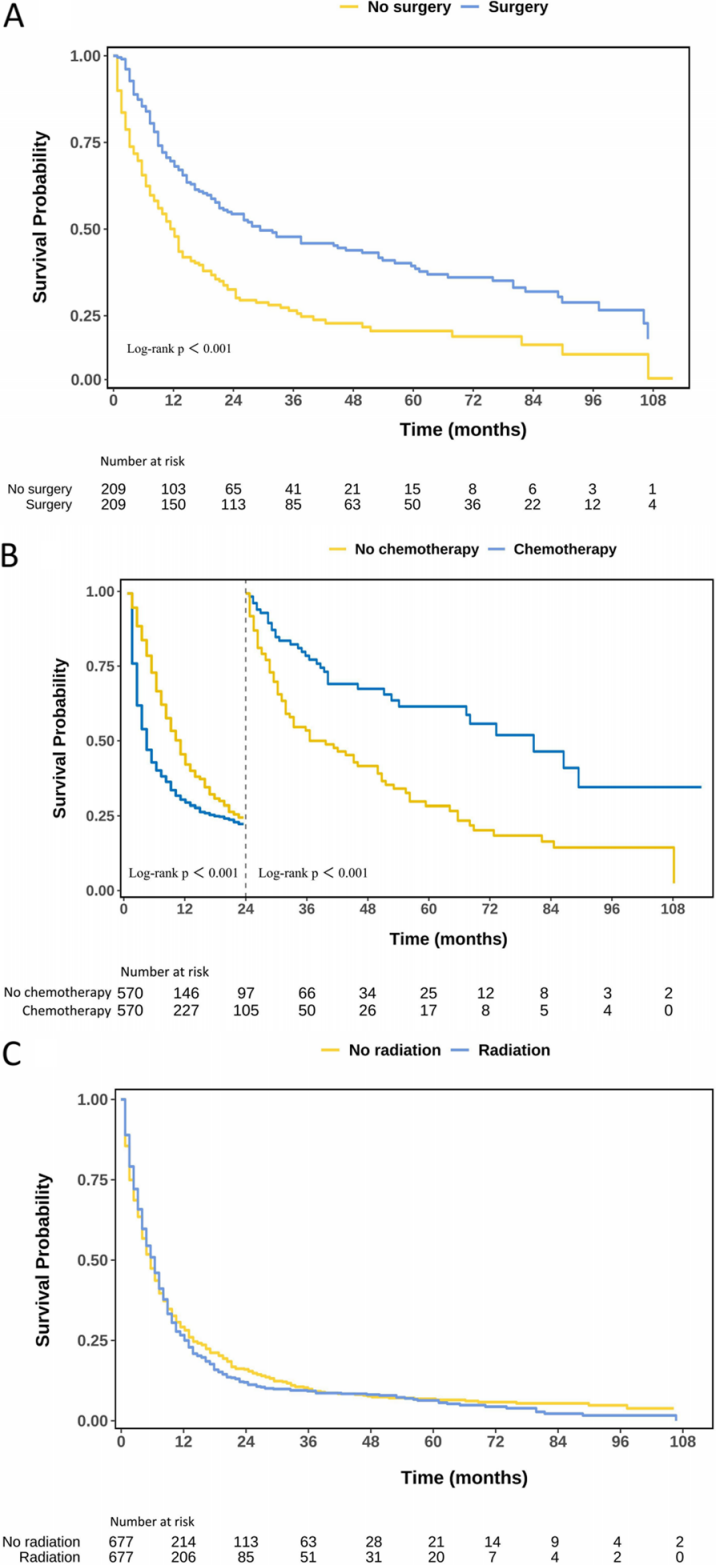
KM curves of mNSCLC-NET patients with each treatment modality after PSM. **A** KM curve of OS in surgery and non-surgical treatment groups. **B** KM curve analysis of survival rates between chemotherapy and non-chemotherapy patients based on the 2-year landmark time point. **C** KM curve of OS in radiotherapy and non-radiotherapy treatment groups

Following PSM, the mOS was comparable between the surgical and non-surgical groups, the radiotherapy and non-radiotherapy groups, and the chemotherapy and non-chemotherapy groups, aligning with the trends observed prior to PSM. Moreover, KM analysis reaffirmed that both surgical intervention and chemotherapy could significantly enhance patients’ survival probability (*P* < 0.05; Fig. 3). Landmark survival curve analysis showed that chemotherapy improved the survival rate of lung cancer patients in the first 24 months of treatment, with a mOS of 10 months in the chemotherapy group (4 months in the non—chemotherapy group). However, after 24 months, patients receiving chemotherapy had a worse prognosis (*P* < 0.05), and the 36—month survival rate dropped to 16.60% in the chemotherapy group (21.85% in the non—chemotherapy group).

### Evaluation of different treatment methods

**Fig. 4:**
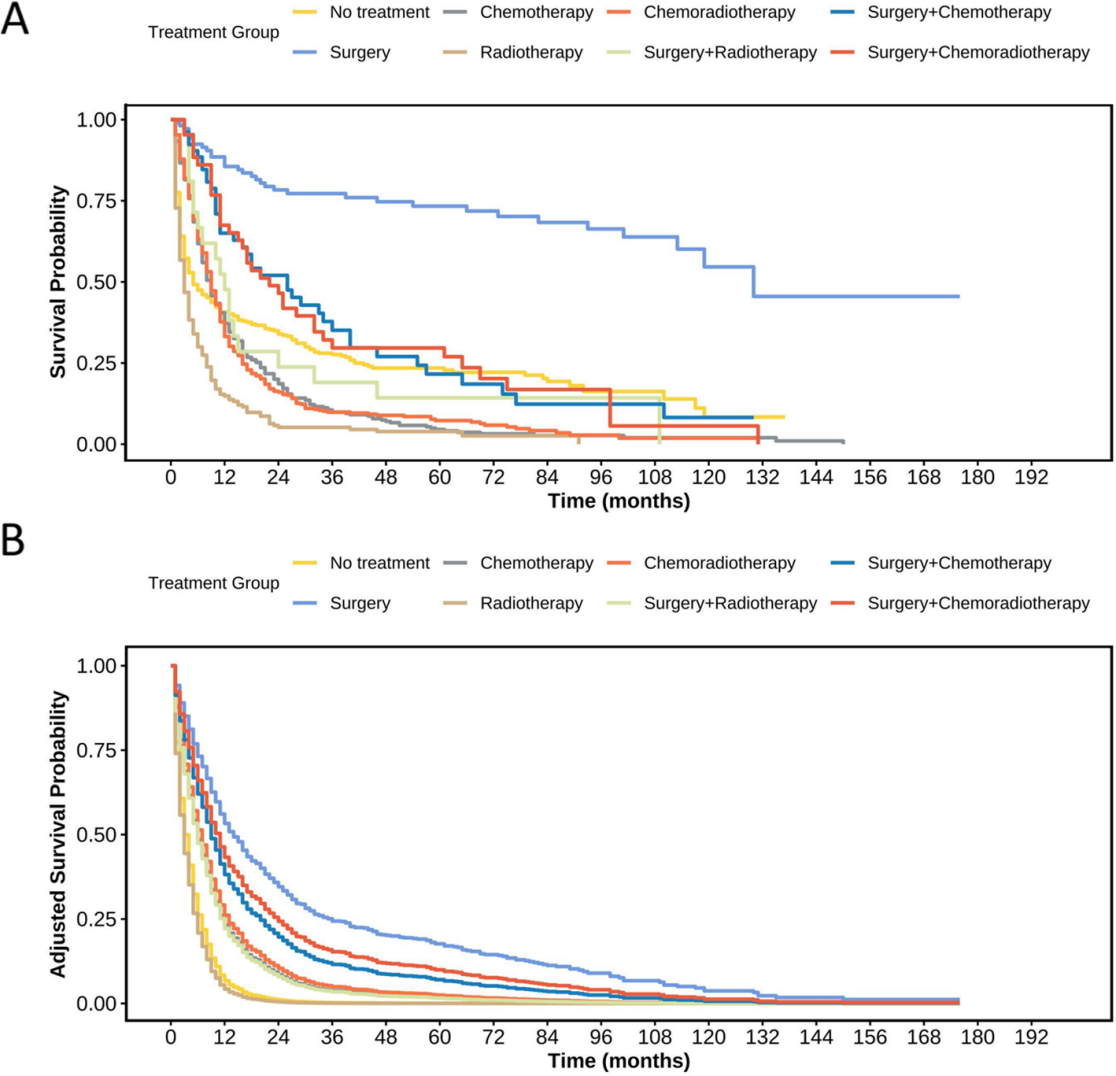
KM curves for mNSCLC—NET in different treatment modalities. **A** KM curves of OS before adjustment; **B** KM curves of OS after adjustment with adjustments made for age, sex, histology, T stage, N stage

To compare the effects of different treatment modalities on patients’ prognosis, we grouped patients into eight categories according to treatment: no—treatment (patients received no radiotherapy, chemotherapy, or surgery after diagnosis); surgery only; chemotherapy only; radiotherapy only; chemoradiotherapy; surgery + chemotherapy; surgery + radiotherapy; and surgery + chemoradiotherapy. Characteristics of mNSCLC—NET patients are presented in Table S5. The Cox regression model was used to adjust for potentially unbalanced confounding factors (Table S6). The survival curves are shown in Fig. 4A, and the adjusted curves in Fig. 4B. Median survival times (95% CI) for different treatments of mNSCLC—NET are listed in Table 2. Compared with the untreated group, surgical patients had the best OS (mOS: 130 months, Log—rank *P* < 0.001; adjusted HR 0.23, 95% CI 0.16—0.33, *P* < 0.001). For some patients with mNSCLC—NET who missed the opportunity for surgical treatment at initial diagnosis, chemotherapy can improve the short—term prognosis to some extent. However, the long—term toxic effects and tumor resistance associated with chemotherapy cannot be ignored, as these factors may have an adverse impact on the overall treatment outcome and long—term quality of life of patients.

**Table 2:** The median survival (95% CI) of different treatment modalities in mNSCLC—NET

Therapy methods	mOS (95%CI)	*P* value*
No—treatment	5.00 (4.00—9.00)	
Surgery	130.00 (113.00—NA)	< 0.001
Chemotherapy	9.00 (8.00—10.00)	0.009
Radiotherapy	3.00 (3.00—4.00)	< 0.001
Chemoradiotherapy	9.00 (8.00—10.00)	0.004
Surgery + chemotherapy	12.00 (6.00—32.00)	0.899
Surgery + radiotherapy	26.00 (16.00—40.00)	0.008
Surgery + chemoradiotherapy	22.00 (15.00—34.00)	0.025

*Abbreviations*: *mNSCLC-NET* metastatic non-small cell neuroendocrine tumor, *mOS* median overall survival

^*^Compared with No—treatment group, Log-rank

### Model construction and evaluation

**Fig. 5:**
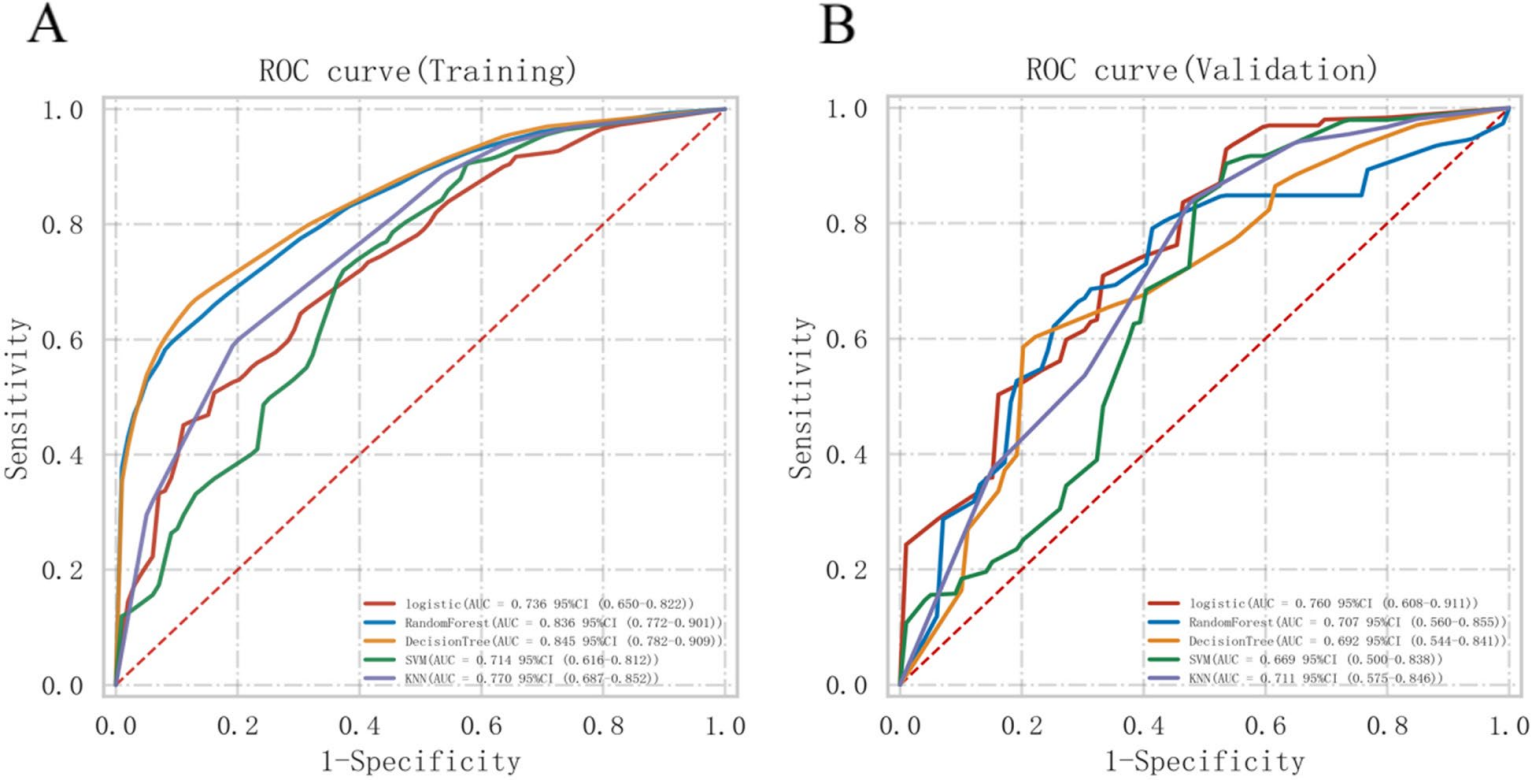
Receiver operating characteristic curves of five machine learning classifiers for the prediction of potential candidates among mNSCLC-NET patients likely to benefit from PTR in the training (**A**) and validation sets (**B**)

**Fig. 6:**
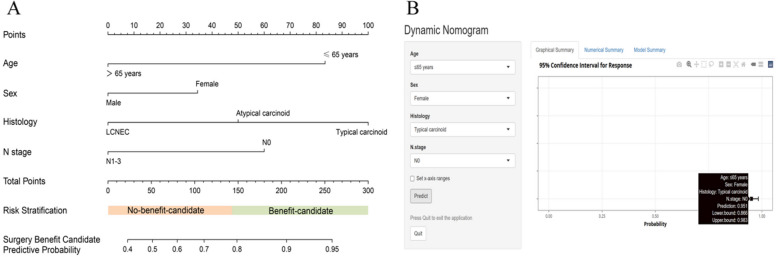
**A** Nomogram for predicting potential candidates likely to benefit from PTR among mNSCLC-NET patients. A logistic regression algorithm was used to establish a nomogram. The final score (i.e., total score) was calculated from the sum of the individual scores of the four variables in the nomogram. **B** To assist researchers and clinicians, an online version of the nomogram is available at https://mnsclc-net.shinyapps.io/benefitPTR/

These findings indicated that some mNSCLC-NET patients benefit from PTR. Among those who underwent surgery, 71.7% (150) who survived over 11 months were deemed to benefit from surgery, whereas the others were classified as nonbeneficial. We developed and validated a predictive model using the surgery group by randomly dividing patients into a training set (70%) and a validation set (30%), with no notable differences between them (Table S7). Based on the Boruta algorithm, four variables were selected as input variables for the models (Figure S2). In the training cohort, the DT had the highest AUC of 0.845. In the test cohort, LR had the highest AUC of 0.760 (Table 3). Additionally, Fig. 5 compares the ROC curves of the five ML classifiers in the training and validation sets, while Table S8 presented the AUC values and p values between each pair of classifiers obtained from DeLong’s test. Consequently, the LR model was selected as the final model. Furthermore, calibration plots and DCA were constructed to further validate the performance of the LR model (Figure S3). Finally, the LR model was converted into a nomogram for better understanding and utilization (Fig. 6A). An online nomogram at https://mnsclc-net.shinyapps.io/benefitPTR/ helps researchers and clinicians identify mNSCLC-NET patients who might benefit from PTR by predicting surgical benefit via clinical variables (Fig. 6B).

**Table 3:** Diagnostic performance of five machine learning classifiers in training and validation sets

Models	**Training set**				**Validation Set**			
	AUC (95% CI)	ACC	SEN	SPE	AUC (95% CI)	ACC	SEN	SPE
LR	0.736 (0.650–0.822)	61.3%	52.9%	79.8%	0.760 (0.608–0.911)	61.9%	59.5%	72.3%
RF	0.836 (0.772–0.901)	68.2%	58.4%	91.8%	0.707 (0.560–0.855)	54.8%	44.0%	85.1%
DT	0.845 (0.782–0.909)	69.9%	61.7%	91.2%	0.692 (0.544–0.841)	61.9%	56.8%	74.2%
SVC	0.714 (0.616–0.812)	68.2%	74.2%	54.5%	0.669 (0.500–0.838)	69.8%	77.4%	49.5%
KNN	0.770 (0.687–0.852)	25.7%	NA	NA	0.711 (0.575–0.846)	34.1%	NA	NA

*Abbreviations*: *ACC* Accuracy, *AUC* Area under the curve, *SEN* Sensitivity, *SPE* Specificity, *RF* Random Forest, *DT* Decision Tree, *SVC* Support vector machine, *KNN* K-Nearest Neighbor, *LR* logistic regression

**Fig. 7:**
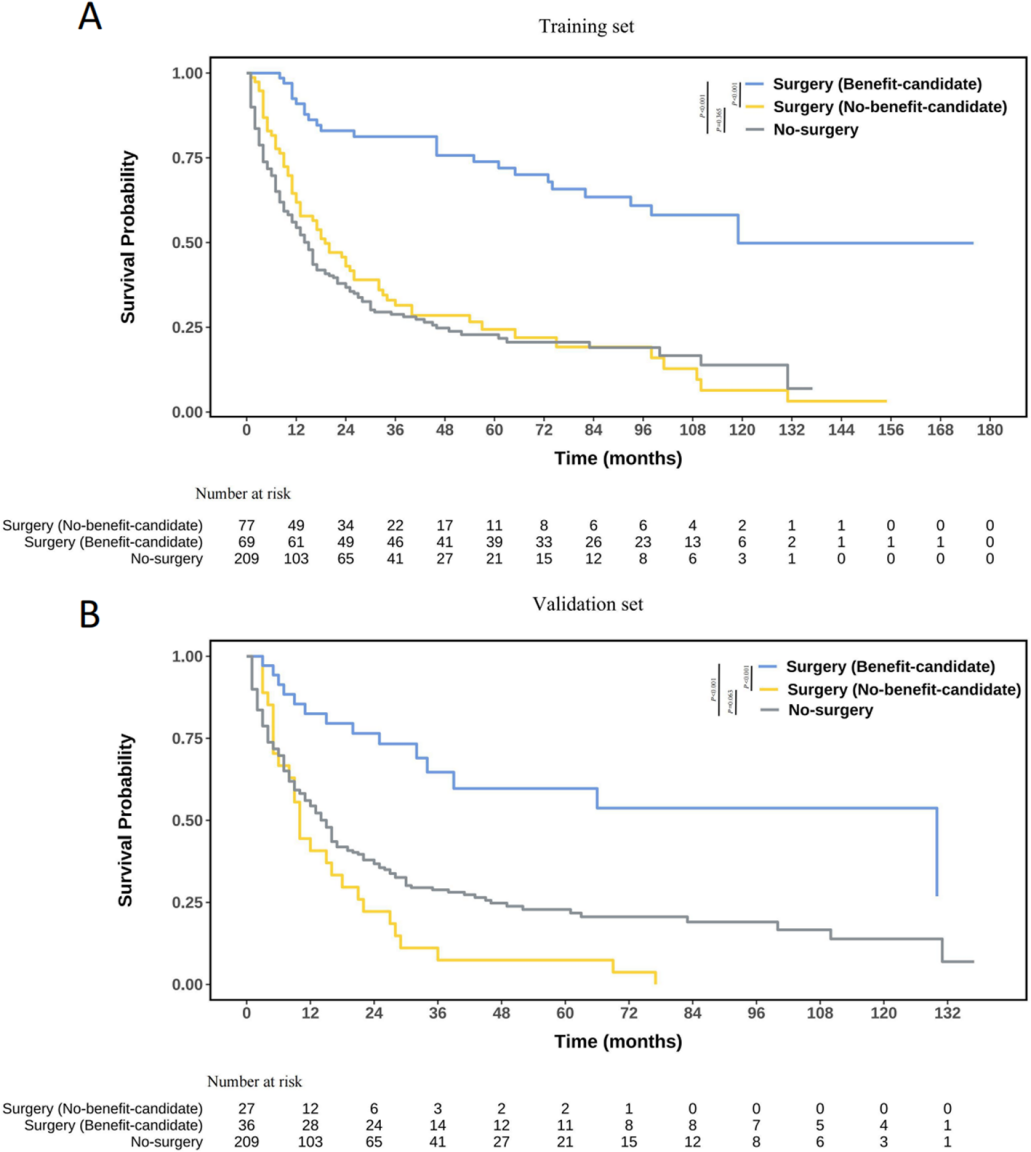
Kaplan‒Meier curves of survival for mNSCLC-NET patients in different benefit classifications according to the nomogram (benefit-candidate and no-benefit candidate groups) and no-surgery group

Using median risk scores, patients were divided into likely and unlikely benefit groups from PTR. K-M analysis in both training and validation datasets showed accurate survival differentiation (Fig. 7). In the validation set, the benefit-candidate group had significantly better OS compared to both the no-benefit-candidate group (HR = 0.189, 95% CI: 0.096–0.374, *P* < 0.001) and the no-surgery group (HR = 0.339, 95% CI: 0.195–0.589, *P* < 0.001). Notably, there was no significant OS difference between the no-benefit-candidate and no-surgery groups (HR = 0.664, 95% CI: 0.440–1.013, *P* = 0.063). This indicates the nomogram effectively identifies mNSCLC-NET patients likely to benefit from PTR and those who may not, suggesting alternative treatments for the latter group.

## Discussion

Currently, it is not yet clear whether PTR provides survival benefits for patients with mNSCLC-NETs, and their management strategies remain contentious. Previous studies have indicated that PTR may have a positive effect on survival rates for these patients. For example, research by Zhang et al., who analyzed 1,763 patients from the SEER database, demonstrated that surgery for mNSCLC-NETs was associated with improved cancer-specific survival in both univariate and multivariate analyses [16]. Another study by Deng et al., which included 2,097 patients, reported that mLCNEC patients who underwent surgical treatment had a significantly longer OS than those who did not (HR 0.456, 95% CI 0.346–0.600, *P* < 0.001) [17]. However, international guidelines and consensus statements do not explicitly recommend performing surgery for primary NSCLC-NETs typically during the metastatic phase [3]. Advancements in preoperative care, surgical techniques, and systemic treatments, along with greater focus on clinical decision-making by multidisciplinary teams, have led to a reevaluation of surgical intervention for mNSCLC patients [18, 19]. Our study comprehensively explored treatment outcomes in patients with mNSCLC—NETs. It first demonstrated that surgery on the primary tumor significantly boosted survival rates. The 5—year survival rate in the surgery group was 47.3%, compared to a mere 9.5% in the non—surgery group. Even after 1:1 PSM, surgical patients still had superior survival outcomes. When comparing different treatment modalities, we found stage—specific efficacy of chemotherapy. Chemotherapy achieved favorable short—term survival benefits, but its effectiveness declined after 24 months. This was likely due to two factors: tumor cell resistance weakening drug efficacy and long—term drug toxicity harming patients’ health and treatment progress. In contrast, surgical treatment outperformed traditional chemotherapy, with a median mOS of 130 months. These findings suggest that contrary to the general belief that PTR is suitable only for early-stage NSCLC-NETs, PTR plays a crucial role in improving the prognosis of patients with mNSCLC-NETs. This study revealed no substantial difference in survival rates between nonbeneficial candidates who underwent surgery and those who did not (*P* > 0.05), highlighting that surgery may not provide benefits for all candidates. Therefore, we suggest that for patients with mNSCLC-NETs, PTR should neither be simply rejected nor universally recommended. Instead, it should be decided on a case-by-case basis, with careful patient selection to optimize survival outcomes for certain individuals.

This study revealed that N stage, histological features such as, sex, and age are crucial factors for predicting whether mNSCLC-NET patients can benefit from PTR. These findings are consistent with previous research [20]. A high N stage is generally associated with poorer postoperative outcomes, likely due to the increased difficulty of surgical clearance. Although the TNM staging system is an important tool for assessing survival rates and planning treatment for lung cancer patients, individualized factors such as histological type, age, and sex also significantly impact cancer survival rates [21]. Therefore, it is essential to develop an improved staging prediction system that incorporates these individualized factors. The histological type is an important clinical predictor of surgical benefit. NSCLC-NETs include low-grade TA, intermediate-grade AC, and high-grade LCNEC [22]. Although all these cancers originate from neuroendocrine cells, high-grade tumors have a greater capacity for proliferation and metastasis, leading to a poorer prognosis [2]. Age is also a clinical predictor of surgical benefit. This finding is consistent with previous research, which indicates that younger patients generally experience better postoperative outcomes following surgery [23]. This may be because older patients often have more comorbidities and fewer opportunities for an effective cure [24]. Sex is an important clinical factor for predicting surgical benefit, which is consistent with previous research [25]. This may be because the expression of estrogen and progesterone receptors is related to tumor cell growth in neuroendocrine tumor patients, with receptor-negative tumors generally having a poorer prognosis [26].

In this study, we trained and validated seven ML models to predict postoperative benefit in patients with mNSCLC-NETs. We used methods such as the DeLong test to compare model performance and found that the LR model exhibited superior predictive capability. In addition, we developed a web-based nomogram that effectively predicts whether mNSCLC-NET patients benefit from PTR. The prognostic factors used in the nomogram are straightforward, easily accessible, and highly actionable.

However, there are several limitations to this study. First, as a retrospective study, potential selection bias may have impacted our findings. Second, the SEER database frequently lacks comprehensive details on genetic mutations, tumor markers, and the characteristics and spread of metastatic disease, along with a dearth of information on the postoperative quality of life of surgical patients. Finally, because the SEER database comprises only U.S. patients, the applicability of our results to a global population might be restricted. Therefore, the establishment of future high-quality cancer databases in China and other countries would facilitate direct cross-regional comparisons. Meanwhile, additional research through well-designed prospective randomized trials is necessary to better understand the impact of PTR interventions on mNSCLC-NET patients.

## Conclusions

This study revealed that PTR prolongs survival time in mNSCLC-NET patients. On the basis of easily accessible data, our nomogram can identify which mNSCLC-NET patients may benefit from PTR. This model provides clinicians and patients with a comprehensive assessment of the risks and benefits associated with surgery, offering a new perspective for treatment strategies in this population. Notably, our model does not impose additional financial burdens on patients and holds potential for further research and optimization.

## Supplementary Information

Supplementary Material 1

Supplementary Material 2

## Data Availability

Qualified researchers may request data from the Surveillance, Epidemiology, and End Results databases. The complete details are available at https://seer.cancer.gov.

## Abbreviations

AC: Atypical carcinoid
AJCC: American Joint Committee on Cancer
AUC: Area under the receiver operating characteristic curve
DCA: Decision curve analysis
DT: Decision Tree
ICD-O-3: International Classification of Diseases for Oncology: Third Edition
K‒M: Kaplan‒Meier
KNN: K‒Nearest Neighbor
LCNEC: Large cell neuroendocrine carcinoma
LR: Logistic regression
MDTs: Multidisciplinary teams
ML: Machine learning
mOS: Median overall survival
mNSCLC-NETs: Metastatic non-small cell neuroendocrine tumors
NSCLC: Non-small cell lung cancer
NSCLC-NET: Non-small cell neuroendocrine tumors
OS: Overall survival
PTR: Primary tumor resection
PSM: Propensity score matching
RF: Random forest
SCLC: Small cell lung cancer
SEER: The Surveillance: Epidemiology: and End Results
SVC: Support vector machine
TC: Typical carcinoid
