# Supplementary material for: Primary tumor resection: a new hope or an old illusion for patients with metastatic non-small cell lung neuroendocrine tumors?
Source: World J Surg Oncol. 2025 Oct 31;23:411. doi: 10.1186/s12957-025-04063-y (PMC12577287; doi:10.1186/s12957-025-04063-y)
Supplement: Supplementary file 2 — Supplementary Material 2 [file 12957_2025_4063_MOESM2_ESM.zip › Fig. S1.pdf]

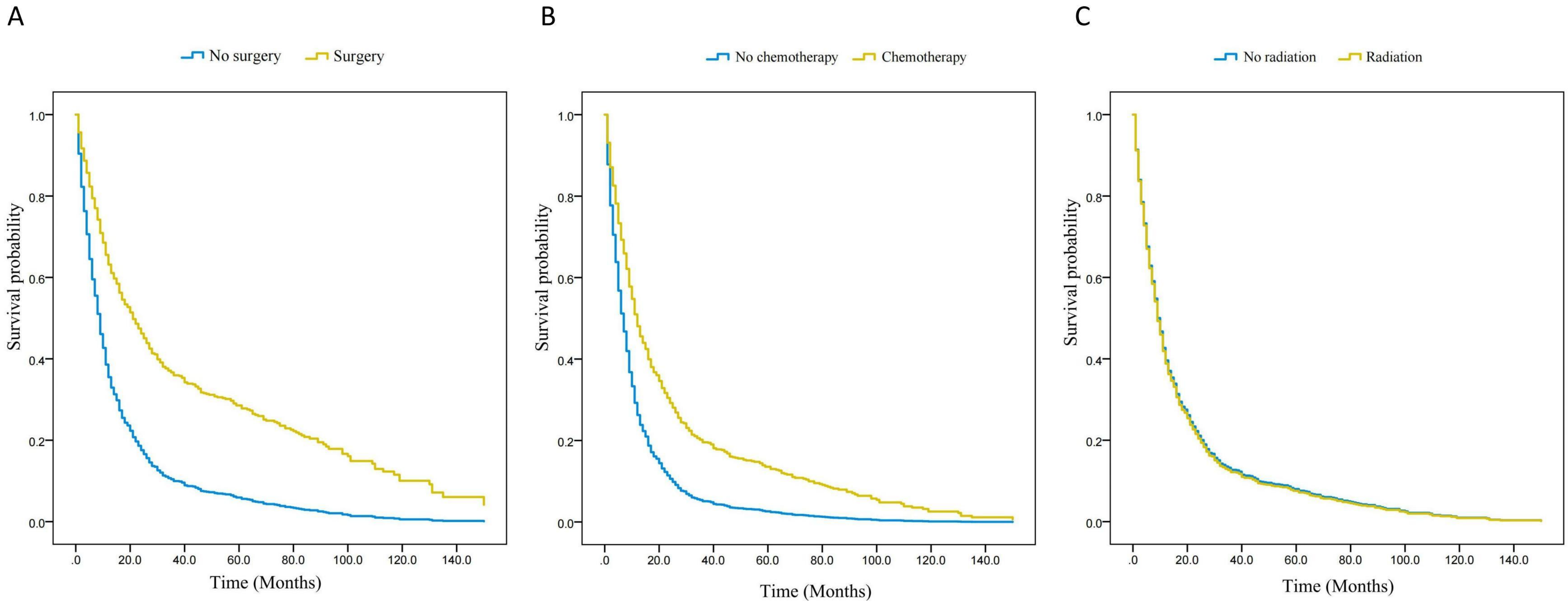

Figure S1: Adjusted KM curves for mNSCLC - NET in different treatment - related groups. A. Adjusted KM curves comparing the surgery group and the non - surgery group for mNSCLC - NET, with adjustments made for age, sex, histology, T stage, N stage, and chemotherapy. B. Adjusted KM curves comparing the chemotherapy group and the non - chemotherapy group for mNSCLC - NET, with adjustments made for age, sex, histology, T stage, N stage, and surgery. C. Adjusted KM curves comparing the radiotherapy group and the non - radiotherapy group for mNSCLC - NET, with adjustments made for age, sex, histology, T stage, N stage, surgery, and chemotherapy.
