# Supplementary material for: Primary tumor resection: a new hope or an old illusion for patients with metastatic non-small cell lung neuroendocrine tumors?
Source: World J Surg Oncol. 2025 Oct 31;23:411. doi: 10.1186/s12957-025-04063-y (PMC12577287; doi:10.1186/s12957-025-04063-y)
Supplement: Supplementary file 2 — Supplementary Material 2 [file 12957_2025_4063_MOESM2_ESM.zip › Fig. S3.pdf]

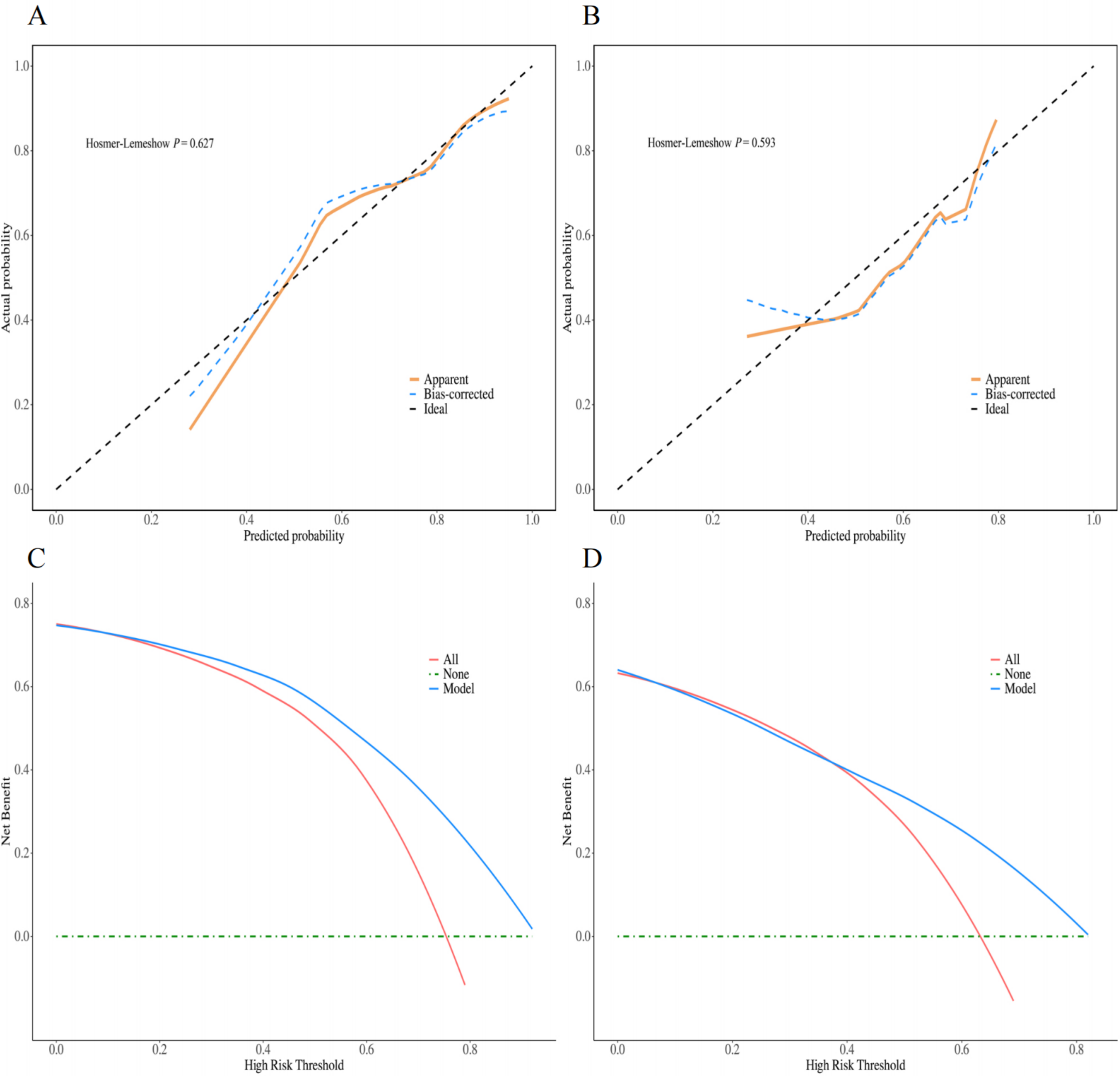

Figure S3: Calibration curve of the nomogram for the training set (A) and the validation set (B). Decision curve analysis of the nomogram for the training set (C) and the validation set (D). A logistic regression algorithm was used to establish a nomogram.
