# Supplementary figures and images for: Primary tumor resection: a new hope or an old illusion for patients with metastatic non-small cell lung neuroendocrine tumors?
Source: World J Surg Oncol. 2025 Oct 31;23:411. doi: 10.1186/s12957-025-04063-y (PMC12577287; doi:10.1186/s12957-025-04063-y)

## Variable importance

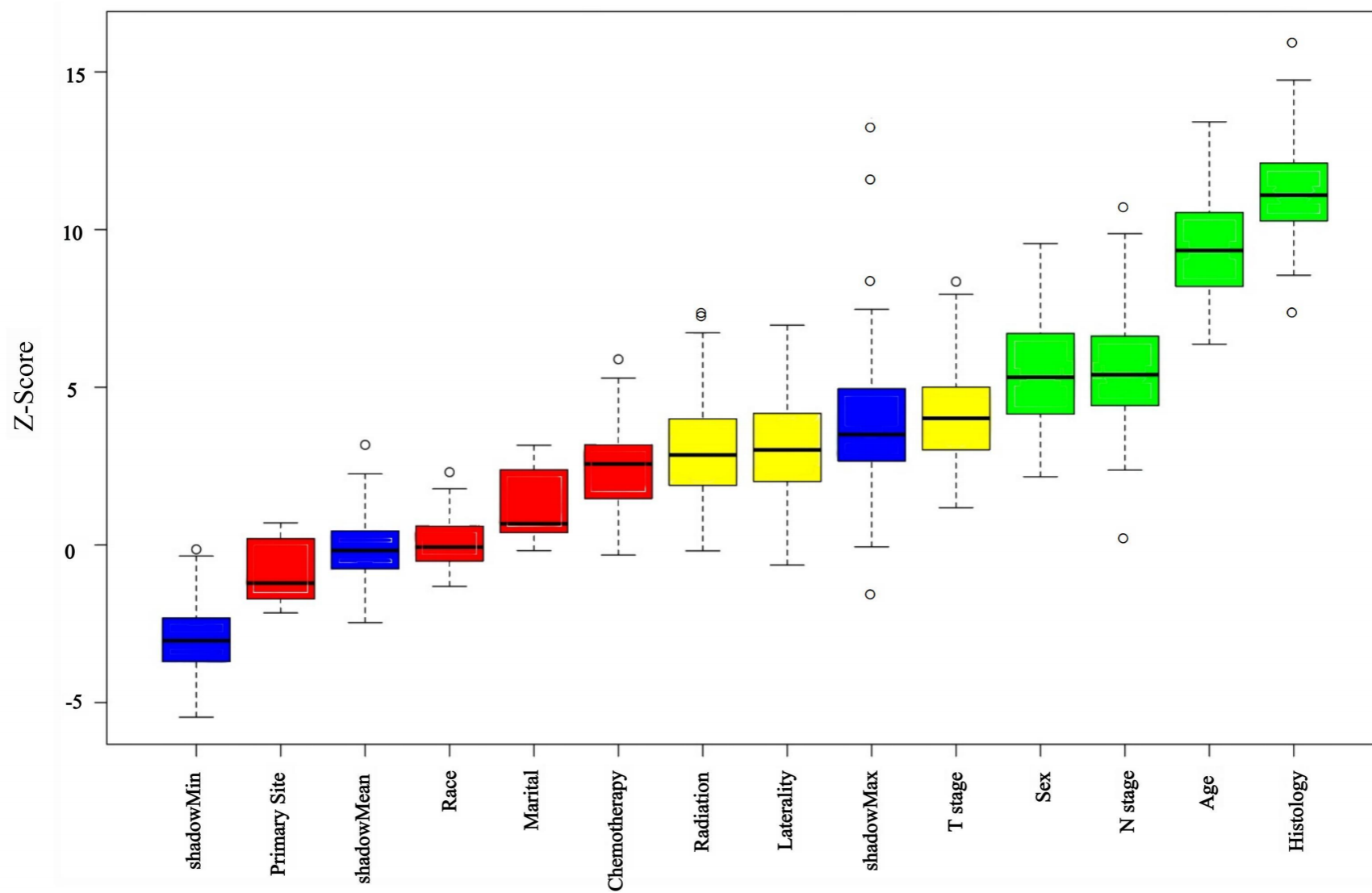

Figure S2: Features selection based on the Boruta algorithm.

Supplement: Supplementary file 2 — Supplementary Material 2 [file 12957_2025_4063_MOESM2_ESM.zip › Fig. S2.pdf]
